# 570 Effectiveness of Hand Edema Management Strategies in the Acute Burn Patient

**DOI:** 10.1093/jbcr/irad045.165

**Published:** 2023-05-15

**Authors:** Renee Warthman, Ari Mehta, Andria Martinez, Claudia Islas, Derek Murray, Kevin Foster, Sierra Williams, Karen Richey

**Affiliations:** Arizona Burn Center at Valleywise Health, Phoenix, Arizona; Arizona Burn Center Valleywise Health, Phoenix, Arizona; Arizona Burn Center at Valleywise Health, Peoria, Arizona; AZ Burn Center at Valleywise Health, Phoenix, Arizona; Arizona Burn Center Valleywise Health, Phoenix, Arizona; AZ Burn Center at Valleywise Health, Cave Creek, Arizona; Legacy Salmon Creek Medical Center, Vancouver, Washington; Arizona Burn Center Valleywise Health, Phoenix, Arizona; Arizona Burn Center at Valleywise Health, Phoenix, Arizona; Arizona Burn Center Valleywise Health, Phoenix, Arizona; Arizona Burn Center at Valleywise Health, Peoria, Arizona; AZ Burn Center at Valleywise Health, Phoenix, Arizona; Arizona Burn Center Valleywise Health, Phoenix, Arizona; AZ Burn Center at Valleywise Health, Cave Creek, Arizona; Legacy Salmon Creek Medical Center, Vancouver, Washington; Arizona Burn Center Valleywise Health, Phoenix, Arizona; Arizona Burn Center at Valleywise Health, Phoenix, Arizona; Arizona Burn Center Valleywise Health, Phoenix, Arizona; Arizona Burn Center at Valleywise Health, Peoria, Arizona; AZ Burn Center at Valleywise Health, Phoenix, Arizona; Arizona Burn Center Valleywise Health, Phoenix, Arizona; AZ Burn Center at Valleywise Health, Cave Creek, Arizona; Legacy Salmon Creek Medical Center, Vancouver, Washington; Arizona Burn Center Valleywise Health, Phoenix, Arizona; Arizona Burn Center at Valleywise Health, Phoenix, Arizona; Arizona Burn Center Valleywise Health, Phoenix, Arizona; Arizona Burn Center at Valleywise Health, Peoria, Arizona; AZ Burn Center at Valleywise Health, Phoenix, Arizona; Arizona Burn Center Valleywise Health, Phoenix, Arizona; AZ Burn Center at Valleywise Health, Cave Creek, Arizona; Legacy Salmon Creek Medical Center, Vancouver, Washington; Arizona Burn Center Valleywise Health, Phoenix, Arizona; Arizona Burn Center at Valleywise Health, Phoenix, Arizona; Arizona Burn Center Valleywise Health, Phoenix, Arizona; Arizona Burn Center at Valleywise Health, Peoria, Arizona; AZ Burn Center at Valleywise Health, Phoenix, Arizona; Arizona Burn Center Valleywise Health, Phoenix, Arizona; AZ Burn Center at Valleywise Health, Cave Creek, Arizona; Legacy Salmon Creek Medical Center, Vancouver, Washington; Arizona Burn Center Valleywise Health, Phoenix, Arizona; Arizona Burn Center at Valleywise Health, Phoenix, Arizona; Arizona Burn Center Valleywise Health, Phoenix, Arizona; Arizona Burn Center at Valleywise Health, Peoria, Arizona; AZ Burn Center at Valleywise Health, Phoenix, Arizona; Arizona Burn Center Valleywise Health, Phoenix, Arizona; AZ Burn Center at Valleywise Health, Cave Creek, Arizona; Legacy Salmon Creek Medical Center, Vancouver, Washington; Arizona Burn Center Valleywise Health, Phoenix, Arizona; Arizona Burn Center at Valleywise Health, Phoenix, Arizona; Arizona Burn Center Valleywise Health, Phoenix, Arizona; Arizona Burn Center at Valleywise Health, Peoria, Arizona; AZ Burn Center at Valleywise Health, Phoenix, Arizona; Arizona Burn Center Valleywise Health, Phoenix, Arizona; AZ Burn Center at Valleywise Health, Cave Creek, Arizona; Legacy Salmon Creek Medical Center, Vancouver, Washington; Arizona Burn Center Valleywise Health, Phoenix, Arizona

## Abstract

**Introduction:**

Hand edema is a common sequalae of burn injuries involving the upper extremity (UE) and larger total body surface area (TBSA) burns requiring fluid resuscitation. Hand edema can be challenging to manage depending on the severity of the injury and, if not adequately addressed, can have long-term functional implications. Multiple edema management techniques are available for therapists treating burn injuries, but little is known about their efficacy. A quality improvement project examined the efficacy of 4 strategies used at our burn center.

**Methods:**

Edema management strategies evaluated included: elevation using a pre-cut foam wedge and pillow, elevation and self-adhesive wrap, elevation and cotton-elastic wrap, and elevation with a chip-bag. Figure-of-8 measurements were taken pre and post intervention. Outcomes included length of time intervention was in place, reason intervention selection, time required to apply intervention, and if intervention was intact prior to removal. Training was completed with staff to ensure consistent application of intervention. Only adult patients were included.

**Results:**

A total of 23 interventions were measured. At follow up, 61% of the interventions were not intact. In each instance this was due to displacement of either the wedge or pillow. Self-adhesive wrap (3.25 cm) followed by cotton-elastic wrap (2.57 cm) in conjunction with elevation achieved the greatest reduction in edema. Elevation of the UE with a wedge and pillow are less effective in managing hand edema alone, with a mean difference of .26 centimeters (cm) decrease in edema. On average, it took therapists less time to apply cotton-elastic wrap (6.4 minutes) vs self-adhesive wrap (14.25 minutes).

**Conclusions:**

In this limited sample, elevation with a pre-cut foam wedge or a pillow was least effective in reducing edema in the hand. Including a secondary edema management technique in conjunction with elevation is more effective in managing hand edema. A larger sample size may benefit increased understanding of edema management techniques within a burn center.

**Applicability of Research to Practice:**

Interdisciplinary training on maintaining edema management strategies is important to promote maintenance of positioning. Further research is warranted to examine the efficacy of hand edema intervention strategies in the acute burn patient.

